# High Neutrophil–Lymphocyte Ratio Predicts Post-stroke Cognitive Impairment in Acute Ischemic Stroke Patients

**DOI:** 10.3389/fneur.2021.693318

**Published:** 2021-07-01

**Authors:** Minwoo Lee, Jae-Sung Lim, Chul-Ho Kim, Sang-Hwa Lee, Yerim Kim, Ju Hun Lee, Min Uk Jang, Mi Sun Oh, Byung-Chul Lee, Kyung-Ho Yu

**Affiliations:** ^1^Department of Neurology, Hallym University Sacred Heart Hospital, Hallym Neurological Institute, Hallym University College of Medicine, Anyang, South Korea; ^2^Department of Neurology, Asan Medical Center, University of Ulsan College of Medicine, Seoul, South Korea; ^3^Department of Neurology, Chuncheon Sacred Heart Hospital, Hallym Neurological Institute, Hallym University College of Medicine, Chuncheon, South Korea; ^4^Department of Neurology, Kangdong Sacred Heart Hospital, Hallym Neurological Institute, Hallym University College of Medicine, Seoul, South Korea; ^5^Department of Neurology, Dongtan Sacred Heart Hospital, Hallym Neurological Institute, Hallym University College of Medicine, Hwaseong, South Korea

**Keywords:** neutrophil, lymphocyte, inflammation, stroke, cognitive impaiment

## Abstract

**Background and Aims:** Systemic inflammation is associated with an increased risk of cognitive impairment and dementia, but the associations between them in stroke patients are less clear. We examined the impact of systemic inflammation represented as the neutrophil-lymphocyte ratio (NLR) on the development of post-stroke cognitive impairment (PSCI) and domain-specific cognitive outcomes 3-month after ischemic stroke.

**Methods:** Using prospective stroke registry data, we consecutively enrolled 345 participants with ischemic stroke whose cognitive functions were evaluated 3-month after stroke. Their cognition was assessed with the Korean version of the Vascular Cognitive Impairment Harmonization Standards and the Korean-Mini Mental Status Examination. PSCI was defined as a z-score of < -2 standard deviations for age, sex, and education adjusted means in at least one cognitive domain. The participants were categorized into five groups according to the quintiles of NLR (lowest NLR, Q1). The cross-sectional association between NLR and PSCI was assessed using multiple logistic regression, adjusting for age, sex, education, vascular risk factors, and stroke type.

**Results:** A total of 345 patients were enrolled. The mean age was 63.0 years and the median NIHSS score and NLR were 2 [1–4] and 2.26 [1.65–2.91], respectively. PSCI was identified in 71 (20.6%) patients. NLR was a significant predictor for PSCI both as a continuous variable (adjusted OR, 1.14; 95% CI, 1.00–1.31) and as a categorical variable (Q5, adjusted OR, 3.26; 95% CI, 1.17–9.08). Patients in the Q5 group (NLR ≥ 3.80) showed significantly worse performance in global cognition and in visuospatial and memory domains.

**Conclusions:** NLR in the acute stage of ischemic stroke was independently associated with PSCI at 3 months after stroke, and high NLR was specifically associated with cognitive dysfunction in the memory and visuospatial domains. Thus, systemic inflammation may be a modifiable risk factor that may influence cognitive outcomes after stroke.

## Introduction

Post-stroke cognitive impairment (PSCI) is a major burden faced by approximately one-third of all stroke survivors (1). As a potentially modifiable risk factor, systemic inflammation has been implicated as an important aspect of cognitive impairment after stroke (2, 3). Systemic inflammation may induce a pro-inflammatory environment in the central nervous system prior to the occurrence of ischemic stroke, which can potentially aggravate the deleterious molecular cascades after stroke (4). Inflammatory markers are implicated in the pathophysiology of Alzheimer's disease (5) and ischemic stroke, (6) but limited data are available on their association with post-stroke cognitive impairment.

The neutrophil–lymphocyte ratio (NLR) is a convenient and readily available parameter of systemic inflammation. High NLR is associated with the prevalence of intracranial atherosclerosis in the neurologically healthy population in a dose-dependent manner (7). NLR of 2.5 and above has also been shown to significantly increase the risk of cerebrovascular and cardiovascular diseases (8). Moreover, NLR higher than 5.0 on admission is an independent factor that increases the risk of hemorrhagic transformation, unfavorable functional outcomes, and mortality after ischemic stroke (9).

In the acute stroke phase, circulating neutrophils are recruited to ischemic lesions and induce destructive cascades, including the production of reactive oxygen species, proteases, and pro-inflammatory cytokines. However, the lymphocyte count relatively decreases in response to stress-induced corticosteroids (10). Therefore, serum NLR may well represent the inflammatory status of the central nervous system in acute ischemic stroke. Nevertheless, previous studies regarding systemic inflammation and PSCI have focused mostly on inflammatory markers such as erythrocyte sedimentation rate (ESR), (2) C-reactive protein (CRP), and interleukins (ILs) (11). However, it is unclear whether NLR is associated with PSCI.

We hypothesized that acute ischemic stroke patients with increased inflammation may be more prone to developing cognitive impairment after ischemic stroke. Thus, we aimed to determine the association between NLR and 3-month cognitive outcomes after acute ischemic stroke using a detailed neuropsychological test battery.

## Methods

### Study Design and Population

This study is a retrospectively conducted observational, cross-sectional study based on a prospective stroke registry. At the time of admission, written informed consent was obtained from all patients or their legal representative to use clinical data in the prospective stroke registry (12). The Institutional Review Board of Hallym University Sacred Heart Hospital has approved this study and exempted the acquisition of additional consent because of its retrospective nature and minimal risk to participants (IRB No.2021-02-010).

Neuropsychological tests were conducted in patients enrolled in the stroke registry who complained of subjective cognitive decline 3 months after stroke onset or at high risk for post-stroke cognitive impairment at the discretion of the attending physician (13). The inclusion criteria for this study were as follows: (i) consecutive patients diagnosed with acute ischemic stroke at the tertiary university hospital from April 2010 to September 2015, (ii) a relevant ischemic lesion observed on diffusion-weighted imaging, (iii) admission within 7 days of symptom onset, and (iv) available data on neuropsychological test results 3 months after stroke onset. Participants with pre-stroke dementia (i.e., those previously diagnosed with dementia or prescribed anticholinesterase inhibitors or memantine) or a premorbid modified Rankin scale score of >2 were excluded from this study. Furthermore, patients who had hearing difficulty, poor cooperation, or neurological deficits such as severe aphasia or motor weakness to a degree that would preclude neuropsychological testing, were excluded.

### Clinical Variables

We reviewed baseline demographic factors, including age, sex, and education level. We also evaluated vascular risk factors including hypertension, diabetes mellitus, dyslipidemia, atrial fibrillation, smoking status, and previous history of stroke. Clinical factors, including initial stroke severity and stroke subtype, were also assessed. Initial stroke severity was assessed using the National Institutes of Health Stroke Scale (NIHSS) score, and stroke subtypes were classified according to the Trial of Org 10172 in Acute Stroke Treatment (TOAST) classification (14). Blood samples for complete blood count were collected in calcium EDTA tubes on arrival at the emergency room and centrifuged (2000 rpm for 20 min at 4°C). Then, the complete blood cell counts were analyzed using an auto-analyzer (XN-3000, Sysmex, Kobe, Japan). The NLR was calculated by dividing the relative neutrophil percentage by the relative lymphocyte percentage (15).

All participants performed brain magnetic resonance imaging, performed using a 3T whole-body magnetic resonance imaging system. The location and number of acute ischemic stroke lesions were assessed and quantified using diffusion-weighted images. Stroke lesions were subdivided as follows: (i) cortical *vs*. subcortical only, (ii) left vs. right vs. both, and (iii) single vs. multiple (16).

### Neuropsychological Evaluations

Participants completed a 60-min neuropsychological test using the Korean version of the Vascular Cognitive Impairment Harmonization Standards-Neuropsychological Protocol (K-VCIHS-NP) at 3 months after stroke onset (17). The K-VCIHS-NP evaluates four major cognitive domains—executive/activation, language, visuospatial, and memory functions (18). The executive/activation domain was evaluated using the Korean version of the controlled oral word association test, semantic fluency (animal naming), (19) digit symbol coding, (20) and the Korean version of the trail-making test for elderly patients (21). Tools for other cognitive domains included the Seoul verbal learning test for memory domain, the Korean version of the Boston naming test: short form A for language domain, (22) and the Rey complex figure test copy score for visuospatial domain (23). We also used the Korean version of the Mini-Mental State Examination (K-MMSE) to evaluate general cognitive function (24). All cognitive tests in the K-VCIHS-NP were standardized and validated for use in South Korea, (17) and scores of each cognitive test were transformed to z-scores with adjustment for age, sex, and education level (i.e., normalized scores) for analysis. Domain-specific z-scores of all four major cognitive domains were also assessed. The participants' premorbid cognitive status was assessed with a structured proxy questionnaire using the Korean version of the Informed Questionnaire on Cognitive Decline in the Elderly (IQCODE). Those with IQCODE score over 3.6 were considered to have premorbid cognitive decline (25). The primary outcome was the occurrence of PSCI, which was defined as a z-score of < -2 standard deviations in at least one cognitive domain (26).

### Statistical Analysis

Continuous variables are presented as means ± standard deviation or medians with an interquartile range as appropriate, and categorical variables are presented as frequencies. Baseline demographic and clinical characteristics between the no cognitive impairment group and the PSCI group were compared using Student's *t*-test or the Mann–Whitney *U*-test for continuous variables and the chi-squared test or Fisher's exact test for categorical variables as appropriate. Clinical characteristics among quintiles of NLR were compared using analysis of variance, the Kruskal–Wallis test, or the chi-square test, as appropriate.

Univariable and multivariable logistic regression analyses were performed to investigate the association between NLR and PSCI. Multivariable models were adjusted for variables with a *p* < 0.1 in the univariate analysis and the prespecified variables demographic characteristics (age, sex, and education level), stroke characteristics including initial stroke severity (NIHSS), stroke subtype (TOAST), and lesion characteristics. An association was indicated as the odds ratio or adjusted odds ratio (aOR) with the 95% confidence interval (CI). Analysis of covariance was conducted to compare the z-scores of each domain and global cognitive functions among quintiles of NLR, adjusting for age, sex, education level, and initial stroke severity (NIHSS). All statistical analyses were conducted using SPSS version 26 (SPSS, Inc., Chicago, IL, USA) and R (version 4.0.3; R Foundation for Statistical Computing); two-tailed *p* < 0.05 were considered statistically significant.

## Results

**Figure 1:**
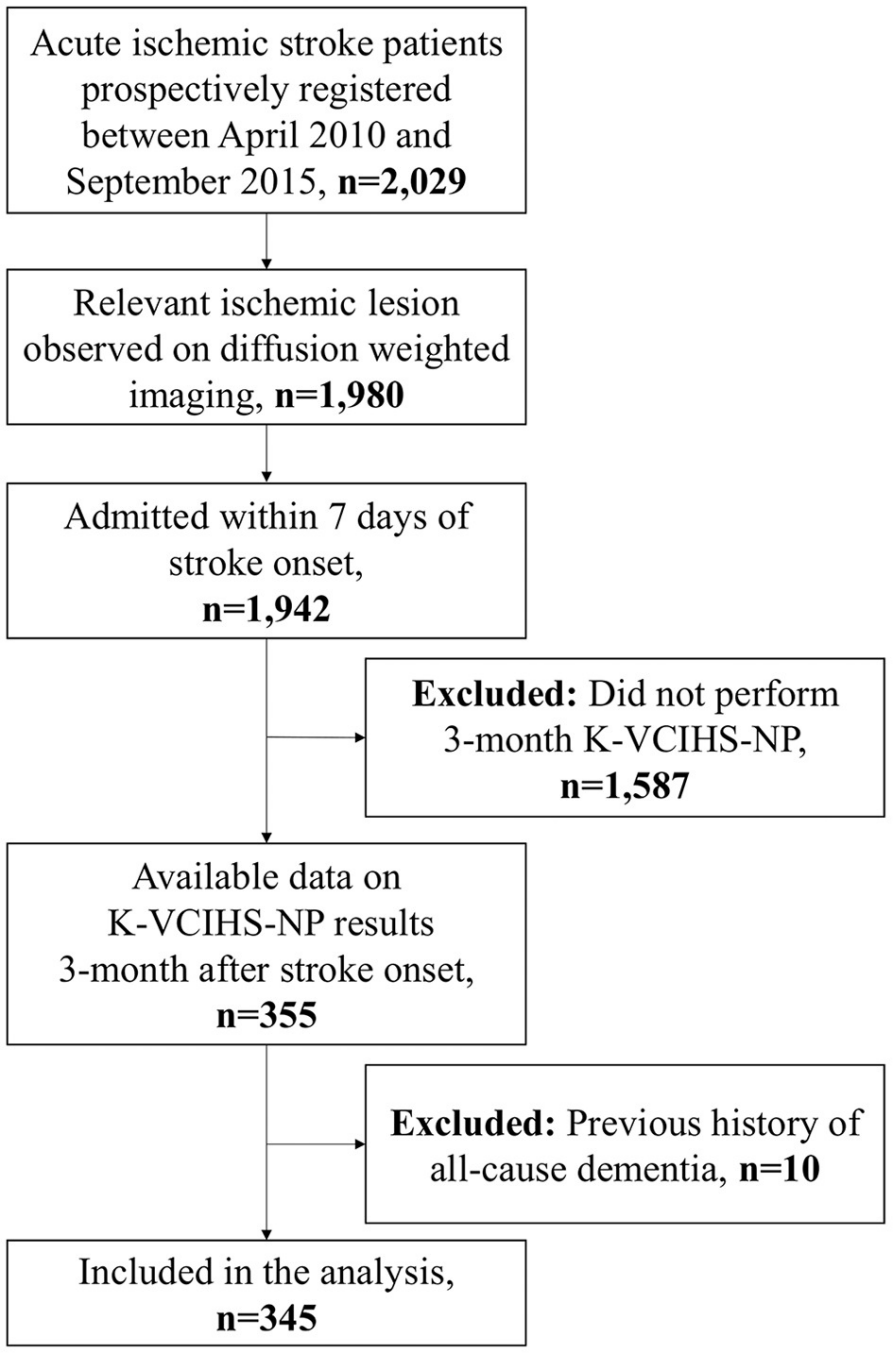
The study flowchart.

Among 2,029 patients included in the inclusion criteria (i) to (iii), 355 patients had available data on the K-VCIHS-NP 3 months after stroke onset (Figure 1). Of these 355 subjects, ten patients were excluded because of pre-stroke history of dementia. Thus, a total of 345 participants who met the eligibility criteria were included in this study. The differences between those included and excluded in this study are demonstrated in the Supplementary Table 1. The mean age was 63.0 years (standard deviation, 12.0 years), and the average interval between stroke onset and performance of a neuropsychological test using the K-VCIHS-NP was 103.4 days (standard deviation, 14.6 days). The median initial NIHSS score and NLR were 2 (interquartile range, 1–4) and 2.26 (interquartile range, 1.65–2.91), respectively. In total, 71 (20.6%) developed PSCI 3 months after ischemic stroke.

**Figure 2:**
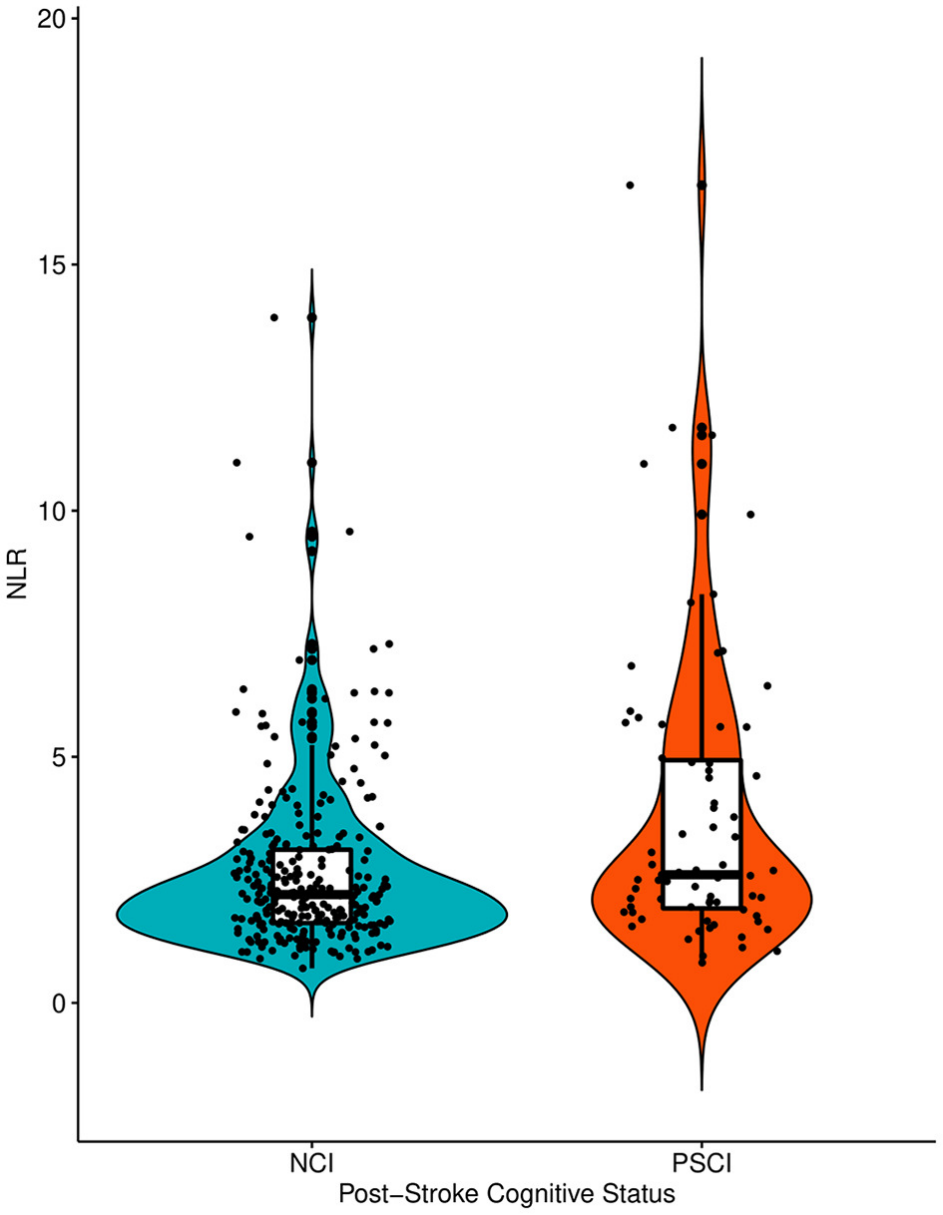
The violin plots demonstrating the differences in the distribution of the NLR levels between NCI and PSCI groups. NLR, Neutrophil-Lymphocyte Ratio; NCI, No Cognitive Impairment; PSCI, Post-Stroke Cognitive Impairment.

The baseline characteristics of the no cognitive impairment and PSCI groups are shown in Table 1. The PSCI group was older and had more women than the no cognitive impairment group. The PSCI group also had a lower frequency of hyperlipidemia, a higher level of fasting blood glucose, higher NIHSS scores on admission, and a higher frequency of large artery atherosclerosis than the no cognitive impairment group. The premorbid cognitive status was not different between the two groups according to the IQCODE scores. The PSCI group had a significantly higher NLR than the no cognitive impairment group (3.9 ± 3.0 vs. 2.7 ± 1.7; *p* = 0.002; Table 1, Figure 2). The comparison of all neuropsychological test results included in the K-VCIHS-NP between NCI and PSCI groups are demonstrated in the Supplementary Table 2. When we stratified the NLR into quintile groups (Q1–Q5), the Q5 group had a higher proportion of large artery atherosclerosis subtype and received thrombolytic therapy more frequently than the other groups. The Q5 group also had the highest frequency of PSCI development (Table 2).

**Table 1:** Baseline characteristics according to the presence of post-stroke cognitive impairment.

	**NCI (*n* = 274)**	**PSCI (*n* = 71)**	***P*-value**
NLR	2.7 ± 1.7	3.9 ± 3.0	0.002
Age, mean ± SD	62.0 ± 12.0	66.7 ± 11.2	0.003
Sex, male, *N* (%)	184 (67.2%)	38 (53.5%)	0.046
Education years, median [IQR]	12.0 [6.0;14.0]	9.0 [6.0;12.0]	0.231
Hypertension, *N* (%)	151 (55.1%)	46 (64.8%)	0.182
Diabetes mellitus, *N* (%)	63 (23.0%)	23 (32.4%)	0.139
Hyperlipidemia, *N* (%)	63 (23.0%)	8 (11.3%)	0.044
Smoking history, *N* (%)	124 (45.3%)	33 (46.5%)	0.96
Atrial fibrillation, *N* (%)	6 (2.2%)	4 (5.6%)	0.252
Coronary artery disease, *N* (%)	14 (5.1%)	5 (7.0%)	0.731
IQCODE >3.6 (*N*, %)	21 (7.7%)	8 (11.2%)	0.645
Initial NIHSS, median [IQR]	2.0 [1.0; 3.0]	3.0 [1.5; 7.5]	0.001
Initial stroke volume (mL), mean ± SD	64.13 ± 169.64	46.17 ± 105.15	0.274
Initial FBS, mean ± SD	139.9 ± 61.3	163.3 ± 69.3	0.006
Stroke subtype (TOAST)			0.003
LAA, *N* (%)	92 (33.8%)	36 (51.4%)	
SVO, *N* (%)	142 (52.2%)	20 (28.6%)	
CE, *N* (%)	12 (4.4%)	8 (11.4%)	
OD, *N* (%)	6 (2.2%)	1 (1.4%)	
UD, *N* (%)	20 (7.4%)	5 (7.1%)	
Subcortical lesion, *N* (%)	51 (18.6%)	11 (15.5%)	0.662
Left hemispheric lesion, *N* (%)	75 (27.4%)	28 (39.4%)	0.067
Multiple lesions, *N* (%)	13 (4.7%)	5 (7.0%)	0.634

*NCI, No cognitive impairment; PSCI, Post-Stroke Cognitive Impairment; NLR, neutrophil-lymphocyte ratio; SD, standard deviation; IQR, interquartile range; IQCODE, Informed Questionnaire on Cognitive Decline in the Elderly; NIHSS, National Institute of Health Stroke Scale; FBS, fasting blood glucose; TOAST, Trial of. Org 10172 in Acute Stroke Treatment; LAA, Large artery atherosclerosis; SVO, Small vessel occlusion; CE, cardioembolism; OD, other determined; UD, Undetermined*.

**Table 2:** Baseline characteristics according to the quintile of NLR.

	**Q1 (≤1.57) *N* = 69**	**Q2 (1.58–2.01) *N* = 69**	**Q3 (2.02–2.55) *N* = 69**	**Q4 (2.56–3.79) *N* = 69**	**Q5 (≥3.80) *N* = 69**	***P*-value**
PSCI, *N* (%)	10 (14.5%)	10 (14.5%)	14 (20.3%)	12 (17.4%)	25 (36.2%)	0.008
Age, mean ± SD	60.9 ± 12.1	62.9 ± 13.4	62.0 ± 10.8	64.9 ± 12.0	64.3 ± 11.4	0.053
Sex, male, *N* (%)	38 (55.1%)	41 (59.4%)	48 (69.6%)	48 (69.6%)	47 (68.1%)	0.250
Education, median [IQR]	12.0 [4.0;13.0]	12.0 [6.0;14.0]	9.0 [6.0;14.0]	9.0 [6.0;12.0]	9.0 [6.0;12.0]	0.673
Hypertension, *N* (%)	35 (50.7%)	37 (53.6%)	34 (49.3%)	42 (60.9%)	49 (71.0%)	0.060
Diabetes mellitus, *N* (%)	16 (23.2%)	16 (23.2%)	20 (29.0%)	12 (17.4%)	22 (31.9%)	0.319
Hyperlipidemia, *N* (%)	14 (20.3%)	14 (20.3%)	13 (18.8%)	13 (18.8%)	17 (24.6%)	0.916
Smoking history, *N* (%)	34 (49.3%)	33 (47.8%)	34 (49.3%)	28 (40.6%)	28 (40.6%)	0.682
Atrial fibrillation, *N* (%)	0 (0.0%)	4 (5.8%)	1 (1.4%)	1 (1.4%)	4 (5.8%)	0.125
Coronary artery disease, *N* (%)	1 (1.4%)	4 (5.8%)	6 (8.7%)	2 (8.7%)	6 (8.7%)	0.215
Stroke subtype						0.001
LAA, *N* (%)	20 (29.0%)	19 (27.9%)	29 (42.0%)	23 (33.3%)	37 (55.2%)	
SVO, *N* (%)	42 (60.9%)	43 (63.2%)	32 (46.4%)	32 (46.4%)	13 (19.4%)	
CE, *N* (%)	2 (2.9%)	2 (2.9%)	4 (5.8%)	5 (7.2%)	7 (10.4%)	
OD, *N* (%)	1 (1.4%)	0 (0.0%)	2 (2.9%)	1 (1.4%)	3 (4.5%)	
UD, *N* (%)	4 (5.8%)	4 (5.9%)	2 (2.9%)	8 (11.6%)	7 (10.4%)	
Initial NIHSS, median [IQR]	2.0 [1.0; 3.0]	1.0 [1.0; 3.0]	2.0 [1.0; 4.0]	2.0 [1.0; 5.0]	3.0 [2.0; 10.0]	0.001
Initial Stroke Volume (mL), mean ± SD	50.24 ± 80.88	65.05 ± 128.28	93.22 ± 287.30	61.96 ± 124.93	30.98 ± 54.51	0.492
Subcortical lesion, *N* (%)	9 (13.0%)	15 (21.7%)	15 (21.7%)	10 (14.5%)	13 (18.8%)	0.547
Left hemispheric lesion, N (%)	25 (36.2%)	15 (21.7%)	18 (26.1%)	26 (37.7%)	19 (27.5%)	0.187
Multiple lesion, *N* (%)	5 (7.2%)	2 (2.9%)	4 (5.8%)	3 (4.3%)	4 (5.8%)	0.822

*NCI, No cognitive impairment; PSCI, Post-Stroke Cognitive Impairment; NLR, neutrophil-lymphocyte ratio; SD, standard deviation; IQR, interquartile range; IQCODE, Informed Questionnaire on Cognitive Decline in the Elderly; NIHSS, National Institute of Health Stroke Scale; FBS, fasting blood glucose; TOAST, Trial of. Org 10172 in Acute Stroke Treatment; LAA, Large artery atherosclerosis; SVO, Small vessel occlusion; CE, cardioembolism; OD, other determined; UD, Undetermined*.

In multiple logistic regression analyses, NLR (aOR, 1.14; 95% CI, 1.00–1.31; Table 3) was a significant predictor for PSCI, after adjusting for age, sex, education level, initial stroke severity, fasting blood sugar level, history of hyperlipidemia, TOAST classification, and presence of left hemispheric lesion. Age (aOR, 1.04; 95% CI, 1.01–1.07), female sex (aOR, 2.27; 95% CI, 1.09–4.73), initial NIHSS score (aOR, 1.08; 95% CI, 1.01–1.16), fasting blood glucose level (aOR, 1.01; 95% CI, 1.00–1.01), hyperlipidemia (aOR, 0.25; 95% CI, 0.10–0.62), large artery atherosclerosis (aOR, 2.59; 95% CI, 1.29–5.21; reference, small vessel occlusion), and left hemispheric lesion (aOR, 1.98; 95% CI, 1.06–3.69) were also significantly associated with PSCI. After conducting additional analysis with quintiles of NLR, the highest quintile (Q5, NLR > 3.8; aOR, 3.26; 95% CI, 1.17–9.08; Table 4) was also significantly associated with an almost 3-fold increased risk of PSCI compared to the Q1 group after adjusting for covariates used in the main analysis.

**Table 3:** Multivariable analysis for possible predictors of PSCI.

	**Crude OR (95% CI)**	***P*-value**	**Adjusted OR (95% CI)**	*P*-value^*^
NLR	1.26 (1.12–1.41)	<0.001	1.14 (1.00–1.31)	0.047
Age	1.03 (1.01–1.06)	0.004	1.04 (1.01–1.07)	0.015
Female sex	1.78 (1.04–3.02)	0.034	2.27 (1.09–4.73)	0.029
Education	0.97 (0.92–1.02)	0.231	1.04 (0.97–1.12)	0.263
Initial NIHSS	1.12 (1.06–1.19)	<0.001	1.08 (1.01–1.16)	0.034
Initial FBS	1.01 (1.00–1.01)	0.007	1.01 (1.00–1.01)	0.018
Hyperlipidemia	0.43 (0.19–0.93)	0.033	0.25 (0.10–0.62)	0.003
**TOAST**				
**(reference SVO)**				
LAA	2.78 (1.52–5.09)	<0.001	2.63 (1.31–5.2)8	0.006
CE	4.73 (1.72–12.99)	0.001	2.18 (0.68–7.01)	0.192
OD	1.18 (0.14–10.34)	0.879	2.22 (0.23–21.83)	0.494
UD	1.77 (0.6–5.26)	0.300	2.28 (0.67–7.72)	0.185
Left hemispheric	1.73 (1.00–2.98)	0.049	1.98 (1.06–3.69)	0.031

* *Adjusted for NLR, age, sex, hyperlipidemia, years of education, initial NIHSS, fasting blood glucose, stroke subtype (TOAST), and left hemispheric lesion*.

**Table 4:** Multivariable analysis for PSCI according to the quintiles of NLR.

**NLR**	**Crude OR (95% CI)**	***P*-value**	**Adjusted OR (95% CI)**	*P*-value^*^
Q1 ( ≤ 1.57)	Ref		Ref	
Q2 (1.58–2.01)	1.00 (0.88–1.14)	0.999	1.32 (0.45–3.88)	0.618
Q3 (2.02–2.55)	1.06 (0.93–1.21)	0.394	1.53 (0.55–4.27)	0.415
Q4 (2.56–3.79)	1.03 (0.90–1.18)	0.670	1.23 (0.44–3.41)	0.693
Q5 (≥3.80)	1.24 (1.09–1.42)	0.002	3.26 (1.17–9.08)	0.024

* *Adjusted for age, sex, hyperlipidemia, years of education, initial NIHSS, fasting blood glucose, stroke subtype (TOAST), and left hemispheric lesion*.

We compared global cognitive functions and all four major cognitive domains among the quintile groups of NLR using analysis of covariance. At 3 months after stroke onset, the z-scores of the K-MMSE were significantly different between the quintile groups after adjusting for age, sex, education level, and initial NIHSS scores (*p* = 0.031). Multiple comparison analysis showed that the Q5 group had the lowest K-MMSE z-scores. The z-scores of visuospatial (*p* = 0.037) and memory function (*p* = 0.049) also differed significantly between the groups. The Q5 group had the lowest z-score of the visuospatial and memory domains (Table 5).

**Table 5:** Comparison of Z scores of cognitive tests according to the quintiles of NLR.

	**Q1 (≤1.57)**	**Q2 (1.58–2.01)**	**Q3 (2.02–2.55)**	**Q4 (2.56–3.79)**	**Q5 (≥3.80)**	*P*-values^*^
K-MMSE	−0.9 ± 2.1	−0.8 ± 1.6	−0.7 ± 1.4	−0.9 ± 2.1	−1.5 ± 2.2	0.031
Frontal	−1.0 ± 1.4	−0.9 ± 1.2	−1.1 ± 1.6	−0.9 ± 1.6	−1.4 ± 1.7	0.113
Visuospatial	−0.8 ± 1.2	−1.2 ± 2.1	−1.2 ± 1.8	−1.2 ± 2.3	−1.5 ± 1.7	0.037
Memory	−0.8 ± 1.3	−0.8 ± 1.2	−0.9 ± 1.0	−1.1 ± 1.5	−1.2 ± 1.4	0.049
Language	−0.2 ± 0.9	−0.0 ± 1.0	−0.1 ± 1.0	−0.2 ± 1.2	−0.3 ± 1.4	0.501

* *Adjusted for age, sex, years of education, and initial NIHSS*.

*K-MMSE, Korean Mini-Mental Status Examination*.

## Discussion

In the current study, we demonstrated that NLR on admission in an acute stroke setting was independently associated with an increased risk of developing PSCI. In addition, the highest quintile (NLR ≥ 3.80) was associated with a 3.26-fold increased risk of PSCI. In ANCOVA analysis, the highest NLR group was associated with global cognitive dysfunction and domain-specific dysfunction in the memory and visuospatial domains.

The findings of this study suggest that an excessive response of the systemic immune system may play a role in the pathomechanisms of PSCI. NLR is a surrogate marker of acute systemic/local inflammation in cardiovascular disorders, including ischemic stroke (10). When an ischemic stroke occurs, the initial inflammatory response cells are recruited to the ischemic area. Among them, neutrophils induce pro-inflammatory mediators, including IL-6, tumor necrosis factor-alpha, and matrix-metalloproteinase-9, and produce reactive oxygen species. This inflammatory cascade leads to disruption of the blood–brain barrier and basal laminar collagen, which may eventually result in an increase in infarct size or development of hemorrhagic transformation (10, 27). In contrast, the neuroprotective subtypes of lymphocytes are decreased in response to stress-induced corticosteroids, leading to poor outcomes after ischemic stroke (28, 29). As a combination of the above two distinct inflammatory markers, NLR is considered as a comprehensive marker of systemic inflammation in acute ischemic stroke patients. Increasing evidence suggests that NLR is an independent predictor of poor prognosis, early neurological deterioration, and mortality after ischemic stroke (9, 30, 31). In general population, the normal range of NLR has been suggested to be between 0.78 and 3.53 (32). The median value of NLR in our study cohort was 2.26 (IQR, 1.65–2.91) and those who developed PSCI had higher NLR compared to the normal cognition group (3.90 vs. 2.70, *p* = 0.002). As our secondary analysis revealed that those with NLR higher than 3.80(Q5) were significantly associated with the development of PSCI, NLR ≥ 3.80 may be a marker for the excessive response of systemic inflammation in stroke population.

Previous studies have shown that systemic inflammation increases the risk of PSCI, irrespective of the lesion size or location (2, 3). In a recent study, ESR had a significant relationship with reduced hippocampal volume and worse cognitive performance after stroke, suggesting persistent pro-inflammatory background as a contributor to hippocampal volume loss (2). Another study using CRP as an inflammatory marker showed that increased levels of serum CRP were associated with global cognitive impairment among post-stroke survivors (3). It is speculated that stimulation of the endothelial intercellular adhesion molecule-1 is mediated by elevated CRP levels and eventually leads to the development of atherosclerosis and, subsequently, cognitive decline (33). As the exact underlying mechanisms is unclear, we hypothesized that secondary brain injury resulting from neutrophil-associated neurotoxicity and decreased host defense by lymphocytes may aggravate neuronal damage and trigger cognitive dysfunction in the recovery phase.

In our study, NLR was primarily associated with lower z-scores in the memory and visuospatial domains. These findings are in line with previous studies revealing that the general population with high CRP levels exhibited impaired cognitive function in memory and visuospatial functions (34). Furthermore, elevated ESR is associated with memory dysfunction and correlates with decreased hippocampal volume (2). A potential explanation is that regional cerebral blood flow in individuals with high CRP and IL-6 levels was decreased in multiple areas linked to memory and visuospatial functions, including the anterior cingulate, precuneus, hippocampus, and parahippocampal gyrus, making these lesions more susceptible to ischemic injury or inflammation (35). Another explanation is in that systemic inflammation is associated with disruption of blood-brain barrier(BBB) integrity, by inducing migration of microglia into cerebral vasculature, which eventually results in phagocytosis of astrocytic end-feet (36). Subtle BBB leakage in the initial stages of stroke or baseline BBB disruption in hippocampal area in the elderly (37) may make BBB of specific regions more vulnerable to systemic inflammation after stroke (38). Thus, patients with high baseline inflammatory marker levels may be more vulnerable to ischemic injury in a domain-specific manner. However, since we did not fully evaluate neuroimaging characteristics, this hypothesis should be investigated in future studies.

This study has several limitations. First, we only evaluated NLR as an inflammatory marker; we did not collect data on other conventional inflammatory markers, including ESR, CRP, and ILs. However, this might provide more unbiased information, considering that all patients with acute ischemic stroke undergo complete blood count analysis on admission. Second, we did not include dynamic changes in NLR values. The combination of admission NLR and dynamic changes would be more comprehensive and may provide better prognostic information. Third, since patients with severe stroke or aphasia who were unable to perform neuropsychological tests were excluded, our results cannot be generalized to all stroke patients. Performing cognitive tests on all stroke patients is not feasible in the real-world practice setting. This inherent bias for attrition has already been discussed, and this should be kept in mind in interpreting the findings of our study (39). Finally, our results could not establish a causal association between NLR and PSCI because of the cross-sectional observational design. The interpretation of our findings is limited to the use of NLR as a potential prognostic and therapeutic target for PSCI in further studies. Despite these limitations, the present study has firstly demonstrated the association between NLR and the development of PSCI after stroke.

## Conclusions

In conclusion, our study demonstrated that NLR in the acute stage of ischemic stroke was independently associated with PSCI at 3 months after stroke. Thus, systemic inflammation may be a modifiable risk factor that may influence cognitive outcomes after stroke. Moreover, cognitive dysfunction was specifically observed in the memory and visuospatial domains. Future clinical trials should clarify whether lowering systemic inflammation by monitoring NLR can also prevent PSCI.

## Data Availability Statement

The raw data supporting the conclusions of this study will be made available from the corresponding authors, upon reasonable request.

## Ethics Statement

The studies involving human participants were reviewed and approved by Institutional Review Board of Hallym University Sacred Heart Hospital. Written informed consent for participation was not required for this study in accordance with the national legislation and the institutional requirements.

## Author Contributions

ML and K-HY: conceptualization. ML and J-SL: methodology. B-CL and MO: validation. ML: formal analysis, investigation, writing—original draft preparation, and supervision. J-SL and K-HY: resources. YK, JL, C-HK, S-HL, and MJ: data curation. J-SL, K-HY, and YK: writing—review and editing. ML and B-CL: visualization. K-HY: Project administration. ML and J-SL: funding acquisition. All authors have read and agreed to the published version of the manuscript.

## Conflict of Interest

The authors declare that the research was conducted in the absence of any commercial or financial relationships that could be construed as a potential conflict of interest.
