# Supplementary material for: High Neutrophil–Lymphocyte Ratio Predicts Post-stroke Cognitive Impairment in Acute Ischemic Stroke Patients
Source: Front Neurol. 2021 Jul 1;12:693318. doi: 10.3389/fneur.2021.693318 (PMC8280279; doi:10.3389/fneur.2021.693318)
Supplement: Supplementary file 1 [file Table_1.DOCX]

| **Supplemental Table 1. Baseline characteristics between those who were included and excluded** | | | | | | | | | |
| --- | --- | --- | --- | --- | --- | --- | --- | --- | --- |
|  | | **Included (n=345)** | | **Excluded (n=1684)** | | | **p-value** | | |
| Age, mean ± SD | | 63.0 ± 12.0 | | 68.4 ± 13.5 | | | <0.001 | | |
| Sex, male, N (%) | | 222 (64.3%) | | 952 (56.5%) | | | 0.009 | | |
| Hypertension, N (%) | | 197 (57.1%) | | 1144 (68.0%) | | | <0.001 | | |
| Diabetes mellitus, N (%) | | 86 (24.9%) | | 605 (35.9%) | | | <0.001 | | |
| Hyperlipidemia, N (%) | | 71 (20.6%) | | 757 (45.0%) | | | <0.001 | | |
| Smoking history, N (%) | | 157 (45.5%) | | 605 (36.0%) | | | 0.001 | | |
| Atrial fibrillation, N (%) | | 10 (2.9%) | | 405 (24.2%) | | | <0.001 | | |
| Coronary Artery Disease, N (%) | | 19 (5.5%) | | 186 (11.0%) | | | 0.003 | | |
| Initial NIHSS, median [IQR] | | 2.0 [1.0; 4.0] | | 3.0 [1.0; 10.0] | | | <0.001 | | |
| Stroke Subtype (TOAST) | |  | |  | | | <0.001 | | |
| LAA, N (%) | | 128 (37.4%) | | 525 (32.1%) | | |  | | |
| SVO, N (%) | | 162 (47.4%) | | 407 (24.9%) | | |  | | |
| CE, N (%) | | 20 (5.8%) | | 409 (25.0%) | | |  | | |
| OD+UD, N (%) | | 32 (9.3%) | | 293 (17.9%) | | |  | | |
| *Abbreviation: NCI; No cognitive impairment, PSCI; Post-Stroke Cognitive Impairment, NLR; neutrophil-lymphocyte ratio, SD; standard deviation, IQR; interquartile range, IQCODE; Informed Questionnaire on Cognitive Decline in the Elderly, NIHSS; National Institute of Health Stroke Scale, FBS; fasting blood glucose, TOAST; Trial of. Org 10172 in Acute Stroke Treatment, LAA; Large artery atherosclerosis, SVO; Small vessel occlusion, CE; cardioembolism, OD; other determined, UD; Undetermined. | | | | | | | | | |
| **Supplemental Table2. Comparison of z-scores and frequency of z-scores less than -2.0 in all the neuropsychological tests performed** | | | | | | | | |  |
|  | **Z-score** | | | | **Frequency of Z-score<=-2.0** | | | |  |
|  | **NCI (n=274)** | **PSCI (n=71)** | **p-value** | | **NCI (n=274)** | **PSCI (n=71)** | | **p-value** |  |
| K-MMSE | -0.4 ± 1.1 | -3.1 ± 2.6 | <0.001 | | 9.60% | 59.60% | | <0.001 |  |
| Frontal | -0.5 ± 1.1 | -3.0 ± 1.2 | <0.001 | | 0.00% | 89.50% | | <0.001 |  |
| Language | 0.1 ± 0.8 | -1.2 ± 1.4 | <0.001 | | 0.00% | 35.10% | | <0.001 |  |
| Visuospatial | -0.7 ± 1.2 | -2.8 ± 2.0 | <0.001 | | 0.00% | 73.70% | | <0.001 |  |
| Memory | -0.7 ± 1.0 | -2.0 ± 1.2 | <0.001 | | 0.00% | 57.90% | | <0.001 |  |
| COWAT_animal | -0.4 ± 0.9 | -1.9 ± 1.0 | <0.001 | | 1.50% | 45.60% | | <0.001 |  |
| COWAT_phonemic | -0.4 ± 0.9 | -1.5 ± 1.0 | <0.001 | | 2.50% | 31.60% | | <0.001 |  |
| DSC | -0.2 ± 1.0 | -1.7 ± 0.7 | <0.001 | | 1.50% | 33.30% | | <0.001 |  |
| TMT-A | -0.0 ± 1.4 | -3.0 ± 3.6 | <0.001 | | 5.60% | 42.10% | | <0.001 |  |
| TMT-B | -0.3 ± 1.1 | -3.8 ± 3.6 | <0.001 | | 7.10% | 61.40% | | <0.001 |  |
| K-BNT | 0.2 ± 0.9 | -1.3 ± 1.5 | <0.001 | | 0.00% | 35.10% | | <0.001 |  |
| RCFT copy | -0.6 ± 1.1 | -3.0 ± 2.0 | <0.001 | | 0.00% | 73.70% | | <0.001 |  |
| RCFT immediate recall | -0.3 ± 0.9 | -1.3 ± 0.8 | <0.001 | | 2.50% | 14.00% | | 0.002 |  |
| RCFT delayed recall | -0.3 ± 1.0 | -1.4 ± 0.7 | <0.001 | | 2.50% | 17.50% | | <0.001 |  |
| RCFT recognition score | -0.3 ± 1.1 | -1.4 ± 1.3 | <0.001 | | 3.00% | 31.60% | | <0.001 |  |
| SVLT immediate recall | -0.6 ± 1.0 | -1.8 ± 1.1 | <0.001 | | 9.10% | 43.90% | | <0.001 |  |
| SVLT delayed recall | -0.7 ± 1.0 | -1.9 ± 1.0 | <0.001 | | 9.10% | 49.10% | | <0.001 |  |
| SVLT recognition score | -0.4 ± 1.1 | -1.6 ± 1.6 | <0.001 | | 7.60% | 33.30% | | <0.001 |  |
| *Abbreviation: NCI; No cognitive impairment, PSCI; Post-Stroke Cognitive Impairment, K-MMSE; Korean version of mini-mental status examination, COWAT; Controlled oral word association test, DSC; Digit symbol coding, TMT; Trail making test, K-BNT; Korean version of Boston naming test, RCFT; Rey complex figure test, SVLT; Seoul-verbal learning test | | | | | | | | |  |
